# Evaluation and implementation of graded *in vivo* exposure for chronic low back pain in a German outpatient setting: a study protocol of a randomized controlled trial

**DOI:** 10.1186/1745-6215-14-203

**Published:** 2013-07-09

**Authors:** Jenny Riecke, Sebastian Holzapfel, Winfried Rief, Julia Anna Glombiewski

**Affiliations:** 1 Department for Clinical Psychology, Psychotherapy University of Marburg, Gutenbergstr 18, Marburg, 35032, Germany

**Keywords:** Fear avoidance, CLBP, Exposure

## Abstract

**Background:**

The purpose of the present study is to introduce an adapted protocol of *in vivo* exposure for fear avoidant back pain patients and its implementation in the German health care system without multidisciplinary teams. Case studies demonstrated promising effects but three preceding randomized controlled trials (RCTs) could not support the former results. More empirical support is necessary to further substantiate the effectiveness of *in vivo* exposure.

**Methods:**

A total of 108 chronic low back pain patients are randomly assigned to one out of three conditions (A: exposure_long (15 sessions), B: exposure_short (10 sessions) or C: control condition cognitive behavioral therapy (15 sessions)). The inclusion criteria are: back pain ≥3 months and a sufficient level of fear-avoidance. An effect evaluation, a process evaluation and an economic evaluation are conducted. Primary outcomes are pain-related disability and pain intensity. Secondary outcomes are: emotional distress, fear avoidance, catastrophizing and costs. Data are collected at baseline, upon completion of the intervention, after 10 sessions, and at six months following completion of treatment. Besides the comparison of exposure *in vivo* and cognitive behavioral therapy (CBT), we additionally compare a short and a long version of exposure to analyze dose response effects.

**Discussion:**

This is, to our knowledge, the first RCT comparing *in vivo* exposure to psychological treatment as usual in terms of cognitive behavioral therapy. Results will help to find out whether a tailored treatment for fear avoidant back pain patients is more effective than a general pain management treatment.

**Trial registration:**

The trial has been registered to ClinicalTrial.gov. The trial registration number is NCT01484418

## Background

Chronic low back pain (CLBP) is one of the most common health problems in Western societies. In Europe, one out of every five people experiences significant back pain that infers with quality of life [1]. In addition to its broad prevalence, CLBP is one of the major causes of medical expenses, work absenteeism and disability [2]. Since cognitive behavioral models of CLBP have become more widely accepted, a large variety of behavioral interventions have become available for the treatment of CLBP [3]. Psychological interventions have been found to be effective, with cognitive behavioral treatments showing moderate to large effect sizes for reducing self-reported pain, pain-related interference, depression and disability [4]. However, it is unknown which interventions work best for particular subgroups of patients [3,5,6] and more research is needed to define subgroups [5]. Vlaeyen and colleagues developed a treatment focusing on fear-avoidant back pain patients [7]. This treatment, known as graded *in vivo* exposure, is based on the fear-avoidance model of chronic pain, which postulates that fear and avoidance of movement contributes to the maintenance of pain via mechanisms of classical conditioning and reinforces disability [8]. As in the treatment of anxiety disorders, the patient is gradually exposed to a feared stimulus, which in the case of CLBP is movement believed to lead to pain or potential injuries to the back. Approximately 10 years ago, the first single case designs showed that *in vivo* exposure reduced pain disability, pain catastrophizing, and pain-related fears with large effect sizes [7,9-11]. In addition to the case studies, three randomized controlled trials (RCTs) have compared graded exposure to a waiting list, graded activity programs [12,13] or treatment as usual, for example, usual medical care [14]. These studies found that *in vivo* exposure was superior to control conditions at reducing pain-related fears and catastrophizing. Only one study found effects (at the trend level) on pain-related disability [14]. In summary, the efficacy of exposure treatment in the RCTs was inferior relative to the preceding case studies.

Therefore, as emphasized by the authors of previously published systematic reviews, additional RCTs, including larger sample sizes, cost-effectiveness-analyses, mediational analyses and treatment fidelity assessments, are required [15-17].

### Problem definition

Several questions concerning exposure *in vivo* treatment (EXP) for chronic back pain remain unanswered:

First, there is a lack of empirical evidence regarding whether interventions tailored for particular subgroups, such as *in vivo* exposure for fear-avoidant pain patients, are more effective than traditional cognitive behavioral treatments for the management of chronic pain problems (CBT-P). Although a variety of definitions of CBT exist, we favor the description provided by Turk, which incorporates behavioral (principles of learning), emotional and cognitive factors. Thus, we attempt to teach our patients “to recognize the connections linking cognitions, affective, behavioral, and physiologic responses together with their joint consequences” [18]. Thus far, *in vivo* exposure has not been compared to cognitive behavioral treatment as it is usually delivered in clinical practice.

Second, it is unknown how many sessions of EXP are needed to achieve sufficient results and that patients get the needed benefit. In a single case paradigm, Vlaeyen *et al*. found significant reductions in disability and pain-related fears after only three exposure sessions [7]. On the other hand, recent reviews indicate that there is evidence that prolonged psychological treatment in chronic pain patients is beneficial [19,20]. Hansen and colleagues showed that between 13 and 18 sessions of therapy in general are required for 50% of patients to improve [21]. Besides the research on the dose–response relationship in psychotherapy in general it is also very important to evaluate and develop the adequate treatment of more specialized treatment approaches. Thus, the analysis of the dose–response relationship in exposure treatment for chronic pain is needed to establish an effective and economical length of treatment.

Third, CLBP is often described as a socio-economic problem because it is a major cause of medical expenses, work absenteeism and disability [2]. However, economic aspects of CLBP treatments (for example, cost-effectiveness) have generally received little attention and thus should be incorporated into RCTs [5,22].

### Objectives

The present investigation, which began recruitment in August 2011 and is currently ongoing, utilizes a three-arm randomized controlled trial method to assess the efficacy of graded *in vivo* exposure (EXP) for CLBP relative to cognitive behavioral therapy (CBT-P). To our knowledge, this is the first study to implement *in vivo* exposure for CLBP patients in an outpatient setting in Germany, and is also the first study to compare graded *in vivo* exposure to CBT. Accordingly, we will assess the feasibility of this new treatment approach. Dose-effects will be analyzed by comparing a short and a long version of EXP. In addition, cost-effectiveness will be evaluated. Besides, the main questions meditational analyses and examination of effects of generalization are planned.

#### Research questions

1) Is EXP more effective (at post-treatment and at six-month follow-up) at reducing pain-related disability and pain intensity (primary outcomes) as well as other symptoms compared to CBT-P in patients with fear-avoidance beliefs?

2) Are there any differences in treatment effects between the short (10 sessions) and the long (15 sessions) versions of exposure at post-treatment and at six-month follow-up?

3) What is the cost-effectiveness of EXP as compared to CBT-P?

## Methods

### Design

A three-arm randomized controlled clinical trial will be performed in a university-based outpatient clinic in Marburg (Psychotherapieambulanz der Universität Marburg, PAM), Germany.

Patients are assigned to one of three conditions (A: exposure_long; B: exposure_short or C: control condition, specifically cognitive behavioral therapy for the management of chronic pain).

The study includes assessments at baseline, mid-treatment, post-treatment and six-month follow-up, as well as process measures.

The study was approved by the ethics committee of The German Association of Psychology (DGPS, WR 052010_1).

### Participants

Our target sample is 108 patients with CLBP. Potential study patients will be recruited via advertisements in local newspapers, doctors´ offices and from the waiting list of our outpatient clinic. Potential participants are screened through an initial phone interview with a research assistant examining the basic inclusion criteria. Those who fulfill all of the basic criteria are invited to a second screening in our outpatient clinic where the criteria of fear-avoidance and disability are assessed.

### Inclusion and exclusion criteria

Patients with CLBP are included if they meet all of the following criteria:

1. Basic criteria:

1.1 Chronic low back pain for at least three months.

1.1 Age: 18 to 66.

2. Additional criteria:

2.1 TSK (Tampa Scale of Kinesiophobia) [23] ≥35 or Phoda-Profile (Photo Series of Daily Activities) [24]: harm ratings of 13 activities >50, including 8 >80 (range 0 to 100, with 0 = “not harmful at all” and 100 = “extremely harmful for my back”).

2.1 Sufficient level of disability, as defined by QBPDS ≥15 (Quebec Back Pain Disability Scale) [25].

Exclusion criteria include: back surgeries during the last six months or planned surgeries, Red Flags [26], inability to read or write in German, pregnancy, alcohol addiction, psychotic disorders and current psychological treatment. Patients are excluded if they are unable to attend sessions regularly for physical or psychological reasons.

Physical and psychological comorbidities, such as depression, are not exclusionary so long as patients are able to attend sessions and complete homework. Recruitment started in August 2011 and will continue until the target sample of 108 patients with CLBP is achieved (expected to occur near the end of 2013). We anticipate that this timeframe will be adequate to achieve recruitment goals.

### Sample size calculation

To identify the target number of participants we used the G-Power Analysis software program (University of Düsseldorf, Germany http://www.psycho.uni-duesseldorf.de/abteilungen/aap/gpower3.) [27]. Based on the effects of previous RCTs, we expected a small effect group x time (repeated measures) of 0.2 for the comparison of three groups (assuming an alpha level = 0.05, two-tailed, β = 0.95, correlation between measurements = 0.5). Results of the power analysis indicated a required sample size of 36 participants per group, for a total of 108. The data will be analyzed with the intent-to-treat (ITT) approach using the last value carried forward and including all patients who intended to participate, regardless of whether they dropped out or not.

### Patient allocation and randomization

Patients are randomized to one of three conditions following a predetermined and computer-generated randomization schedule, pre-stratified by degree of pain catastrophizing (PCS) [28] and disability (PDI) [29]. The median scores of the pain catastrophizing and disability data from a previous study are used as cutoffs. Within each stratum, a randomized block design with a block size of nine is used to ensure equal distribution of important patient characteristics. The randomization procedure is performed by a blinded research assistant who prepares sequentially numbered, sealed, opaque envelops. As is common in psychological treatments, it was not feasible to blind patients or therapists to treatment condition.

### Intervention

We translated and adapted the protocol of Johan Vlaeyen and Jeroen de Jong [7] for implementing EXP in a German outpatient setting. The intervention is delivered by two PhD students who are clinical psychologists and who received two different sessions of training from J. Vlaeyen and J. de Jong. In addition, each session is supervised afterwards by experienced psychologists. The control condition is a cognitive behavioral treatment for pain patients containing basic elements for the management of chronic pain that are standard in our outpatient clinic. The treatment includes graded activity, relaxation (progressive muscle relaxation) and cognitive interventions such as cognitive restructuring (Table 1). Both interventions are offered in a structured, individual setting with weekly appointments of 50 minutes.

**Table 1:** Overview treatment sessions

**Session**	**EXP_long**	**EXP_short**	**CBT_P**
1	Anamnesis	Anamnesis	Anamnesis
2	Psycho-educational part I	Psycho-educational part I	Psycho-educational part I
3	Psycho-educational part II:	Psycho-educational part II:	Goal setting
	Fear-avoidance model	Fear-avoidance model	
4	Fear hierarchy	Fear hierarchy	Behavior analysis
5	Exposure 1	Exposure 1	Graded Activity I
6	Exposure 2	Exposure 2	Graded Activity II
7	Exposure 3	Exposure 3	Health Behavior
8	Exposure 4	Exposure 4	Progressive Relaxation I
9	Exposure 5	Exposure 5	Progressive Relaxation II
10	Exposure 6	Completion	*Cognitive Intervention I:* Attention Shifting
11	Exposure 7		*Cognitive Intervention II:* Identification of automatic thoughts and core beliefs about pain
12	Exposure 8		*Cognitive Intervention III:* Restructuring of dysfunctional thoughts and beliefs
13	Exposure 9		Individual Session I
14	Exposure 10		Individual Session II
15	Completion		Completion

CBT-P, Cognitive behavioral treatment for chronic pain problems*;* EXP*,* Exposure treatment.

Treatment sessions are video-recorded and a random selection of videos is evaluated for treatment fidelity. Two blinded raters independently assess protocol adherence, treatment contamination and whether the treatments can be differentiated from each other using the method of assessing treatment delivery in clinical trials (MATD) [30]. In advance, treatment elements of both treatments were identified and listed in a randomized order for the ratings.

#### Common features

Both treatments aim to restore functioning and to decrease pain-related disability. The first two sessions of all treatment conditions are identical. At the intake, participants receive printed material, including background information and worksheets. The first appointment serves as an anamnesis interview which is followed by an educational session. During the educational session, general information about pain, factors related to the maintenance of pain, and physical and physiological changes associated with chronic pain are discussed. Finally, all patients are encouraged to develop feasible treatment goals with respect to activities in which they would like to re-engage.

#### EXP - unique features

Graded *in vivo* exposure (Table 1) aims to reduce pain-related disability via overcoming fear of pain/movements. The program offers two versions of exposure: a long form including 15 sessions (10 exposures) and a short form including 10 meetings (5 exposure sessions). After the first two sessions, the patient is encouraged to develop an individualized fear-avoidance model including pain, pain cognitions and avoidance. This circular model serves as a basis for transferring and explaining the therapeutic rationale. During session four, a fear hierarchy is established using the Photo Series of Daily Activities [31]. The patient makes harm ratings of 100 pictures showing daily activities. If the patient has understood the therapeutic rationale and is willing to do exposure he will be gradually confront feared stimuli, which are movements related to pain or potential injuries of the back. Patients are encouraged to engage in these feared activities as much as possible until anxiety levels have decreased. Behavioral experiments are integrated to challenge catastrophic beliefs about the feared consequences of pain or specific movements. This intervention is intended to produce cognitive and behavioral changes and offers the patient the opportunity to regain trust in his or her body. The process of overcoming fear and learning that feared consequences are unlikely to occur is believed to be the main mechanism of treatment.

#### CBT-P unique features

The primary goal of the CBT approach is the same as that of the exposure treatment, that is, to increase functional capacity. However, the method used to achieve this goal is different. CBT-P encourages patients to develop an adaptive style of coping by maintaining a problem-solving orientation. Patients are provided with different strategies to cope with pain. Initially, patients learn to increase activity by dividing the activities they want to perform in smaller steps to avoid phases of excessive demands followed by long terms of recovery. After this first phase, patients learn to practice progressive relaxation. During the third module, maladaptive pain-related cognitions are identified and discussed. Patients are taught to recognize the links between thoughts, feelings and behavior and to challenge negative appraisals as one strategy for interrupting pain-maintaining circuits. The last two sessions are individualized based on topics relevant to the patient (for example, work-related problems). These topics are discussed with a focus on problem-solving.

An additional table file gives an overview of the treatment sessions in more detail (see Additional file 1, Table 1).

### Outcomes

For outcomes related to pain, we followed the recommendations from the Initiative on Methods, Measurements, and Pain Assessment in Clinical trials (IMMPACT, [32]).

#### Primary outcome measures

Pain-related disability is measured using the Pain Disability Index (PDI) [29,33] and the Quebec Back Pain Disability Scale (QBPDS) [25].

Pain intensity is assessed four times a day using a two-week pain diary comprising an 11-point numeric rating scale (NRS) (0 = no pain, 10 = pain at its worst) and an 11-point scale from the German Pain Questionnaire (Deutscher Schmerzfragebogen,DSF) assess average pain intensity during the past four weeks [34].

#### Secondary outcomes

Fear of movement/pain anxiety is measured using the Tampa Scale of Kinesiophobia (TSK) [23,35] and The Pain Anxiety Symptom Scale (PASS) [36,37]

Pain catastrophizing is assessed using the Pain Catastrophizing Scale (PCS) [28,38].

Emotional distress is measured using the Hospital Anxiety and Depression Scale (HADS) [39,40].

Quality of Life is measured using the EuroQol Questionnaire (EQ-5D) [41,42].

Pain vigilance is measured using the Pain Vigilance and Attention Questionnaire (PVAQ) [43,44].

Physical activity is assessed using the International Physical Activity Questionnaire (IPAQ) [45].

Global perceived effect of treatment is rated on a 7-point scale (1 = completely recovered, 7 = worse than ever) to assess subjective perceptions of recovery.

All instruments have been widely used and demonstrated reliability and validity in the chronic pain population.

#### Tertiary outcomes

The tertiary outcomes include: the Pain Solutions Questionnaire (PASOL) [46], the Psychological Inflexibility in Pain Scale (PIPS) [47], scales assessing coping with pain (Fragebogen zur Erfassung der Schmerzverarbeitung, FESV) [48] and the Pain Self Efficacy Questionnaire (FESS) [49].

#### Additional variables

Several additional variables are assessed in order to examine potential mechanisms of treatment and to assess feasibility and treatment fidelity.

Satisfaction with treatment as a measure of feasibility is evaluated with 10 items on an 11-point scale (0 = strongly disagree, 10 = absolutely agree). In addition, the therapists assessed feasibility in a qualitative manner including a standardized dropout evaluation and assessment of adverse effects.

All therapy sessions are documented within a table, including notes and information about the participants based on evaluations of the therapists or supervision. The therapist also notes when he or she thinks that the assigned treatment approach is not fully adequate for this patient. Furthermore, a standardized dropout evaluation was developed. If a patient breaks off treatment, an independent research assistant asks the participant standardized questions about the reasons for refusing the treatment and possible adverse effects of treatment.

Sessions will be evaluated for treatment fidelity by a research assistant blind to treatment conditions. The fidelity evaluation is based on the approach of Leeuw and colleagues who developed a method of assessing treatment delivery in clinical trials (MATD) [30].

Treatment expectancy and rationale credibility is measured with the credibility and expectancy questionnaire (CEQ) [50].

The therapeutic alliance is measured on an 11-point scale including 13 items (0 = strongly disagree, 10 = absolutely agree).

### Process evaluation

Participants complete a short survey online each week to measure mediating variables relevant to the process of change. The battery includes the TSK, PDI, IPAQ, QBPDS, a numeric rating scale assessing pain severity, and an 11-item scale assessing coping with pain, self-efficacy and avoidance rated on an 11-point scale.

### Economic evaluation

Costs and expenses are evaluated using an adapted version of the short form of the Trimbos/iMTA questionnaire for costs associated with psychiatric illness over the past month (TIC-P) [51]. The total costs include direct health-care and non-health care costs (that is, travel costs, out-of pocket expenses, self-paid medical care), as well as indirect health care costs due to loss of productivity. Direct health-care costs include costs of health-care consumption in general (that is, general practitioner, physical therapists, hospital visits and so on.). The number of consultations is multiplied by the cost of each visit to calculate total costs.

### Data collection

Data are collected at baseline (T0), after 10 sessions (T1, completion of EXP short), upon completion of the intervention (T2), and six months following completion of treatment (T3). Thus, participants in the short version of EXP complete only three assessments. Questionnaires are completed at home via the internet. This procedure reduces missing data and helps to keep study therapists blind to outcome data. Supplemental evaluations are carried out weekly, including process variables. If patients do not have internet access the assessment are provided on a computer in our outpatient clinic. Video recordings are carried out during each session to gather objective data for later analysis of treatment fidelity.

### Data analysis

The latest version of The Statistical Package for Social Science (IBM SPSS Statistics) version 19is used for data analyses. Intention-to-treat analyses (ITT) used to account for deviations from random allocation and missing data. A repeated measures design will be used with primary and secondary measures as dependent variables, group as the between-subjects factor, and time of measurement as the within-subjects factor.

Possible confounding variables (for example, duration of complaints) will be considered and included as covariates according to the results of correlation analyses. Effect sizes (Cohen´s d) will be calculated to determine the magnitude of the mean differences between groups on the outcome variables. According to Cohen´s guidelines, an effect size of .2 indicates a small effect, .5 a medium effect and .8 a large effect [52]. We consider a 30% reduction on the NRS to indicate clinically important change on the core outcome measure of pain intensity [53]. The economic evaluation consisted of a cost-utility analysis and a cost-effectiveness analysis. For both analyses, the incremental cost-effectiveness ratio (ICER) was calculated as (C1-C0)/(E1-E0) where C is the cost and E is the effect and the experimental and comparator conditions are indexed with the 1, 0 subscripts.

The incremental cost-utility ratio will focus on the net costs per quality adjusted life years (QALY) gained. The cost-effectiveness ratio focuses on the net costs per reliable and clinically improved case of disability/pain. Probit analysis will be used for dose–response effects. This approach based on probability theory and includes determining the observed rates and resulting probability of a response for a particular treatment.

### Ethical considerations

Before participating, each patient receives and signs an informed consent form, which describes the details of the intervention and assessments. Participants are informed that they may cease participation at any time without consequences. In addition, participants are offered standard psychotherapy at our outpatient clinic if needed. The Ethics committee of The German Association of Psychology (DGPS, WR 052010_1) approved this trial.

## Discussion

### General

Despite the demonstrated efficacy of cognitive behavioral interventions for the treatment of CLBP [4], research on the efficacy of EXP is lacking [16]. To our knowledge, this is the first study to implement EXP in an outpatient setting in Germany. Furthermore, it is the first randomized clinical trial to evaluate EXP compared to cognitive behavioral treatment as usual for chronic pain. This comparison allows us to investigate whether fear-avoidant back pain patients benefit more from a treatment that addresses their specific pain-related fears than from a general psychological pain management program.

EXP is a relatively new approach for treating CLBP. During the process of developing and testing new interventions, it is important to investigate feasibility, cost-effectiveness and the underlying mechanism of treatment change. A recent review called for additional RCTs investigating approaches to CLBP [15], and with one exception [54], existing published RCTs have not investigated mechanisms of change [17]. To understand the process of change during EXP, the current RCT adds weekly measures of potential key mediators, such as self-efficacy, pain coping and pain beliefs. A novel feature of the current study is the inclusion of a short form of exposure therapy (10 sessions) in addition to a long version comprising 15 sessions. The analysis of the dose–response relationship in exposure treatment is crucial to determining an effective and economical treatment length. Finally, as EXP represents a completely new approach in the German health care system, information about cost-effectiveness is very important. In general, information on the economic aspects of CLBP treatments is lacking [5,22]. Accordingly, we integrated cost-effectiveness analysis with an adapted version of the TIC-P [51].

### Potential strengths of the study protocol

Participants will be recruited over a period of two years, which allows the inclusion of a larger sample (108 participants) relative to a prior RCT (N = 59) [12].

Before administering the intervention, the therapists participated in two workshops taught by Johan Vlaeyen and Jeroen de Jong, the developers of *in vivo* exposure for CLBP. This training ensures that therapists have basic knowledge and competence in implementing the treatment. Weekly supervision is provided, and therapeutic difficulties are discussed as a team. In addition, our research team is in regular contact with the groups in Maastricht and Leuven. Regular meetings allow discussion of specific difficulties with treatment implementation. It has been observed that the quality of treatment is frequently neglected in studies evaluating therapeutic interventions [4,16]. Accordingly, to improve the quality of the treatment protocol, adherence will be assessed through video-recordings of sessions and will be controlled in analyses. The Method of Assessing Treatment Delivery (MATD) proposed by Leeuw and colleagues [30] will be used to assess treatment fidelity.

### Potential limitations of the study protocol

The treatment in the present study is designed for patients with CLBP with sufficient levels of fear and avoidance (TSK ≥35, Phoda-profile), and thus it is unclear whether results are likely to generalize beyond this specific pain population. Nevertheless, we attempted to keep the eligibility criteria very liberal (that is, no exclusion for comorbidities or medication use, moderate level of TSK) to offer broad access to the intervention. Secondly, the present study relies largely on self-report measures.

## Conclusions

It is important to determine whether a specialized treatment is needed for fear-avoidant back pain patients, a high-risk group characterized by substantial impairment, or whether standard CBT programs are adequate? This RCT provides an opportunity to address this question by investigating whether *in vivo* exposure is more effective than psychological treatment as usual for the treatment of CLBP. Furthermore, the results of this study will be used to establish the feasibility and cost-effectiveness of *in vivo* exposure in an outpatient psychological setting.

### Trial status

**Figure 1:**
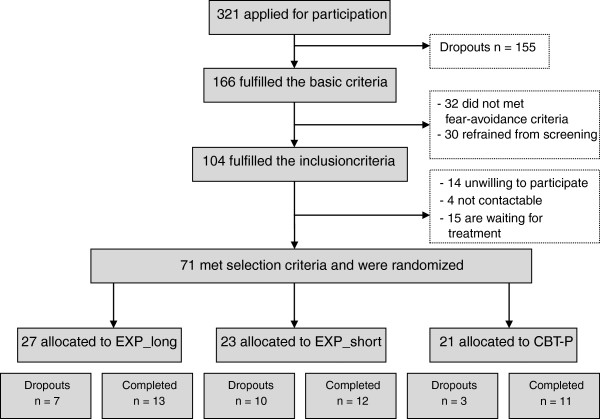
Flow of participants (07/06/2013).

Recruitment of patients started on August 2011 and will proceed until the end of 2013. Data on the effect, process and economic evaluation are expected to be available in 2014. A total of 71 participants had been enrolled and randomized for this trial by May 2013 (Figure 1).

## Abbreviations

CBT-P: Cognitive behavioral treatment for chronic pain; CEQ: Credibility and expectancy questionnaire; CLBP: Chronic low back pain; EQ-5D: EuroQol questionnaire; EXP: Exposure; FESS: Pain self efficacy questionnaire; HADS: Hospital anxiety and depression scale; ICER: Incremental cost-effectiveness ratio; IMMPACT: Initiative on methods measurements, and pain assessment in clinical trials; IPAQ: International physical activity questionnaire; ITT: Intention-to-treat analyses; MATD: Method of assessing treatment delivery in clinical trials; NRS: Numeric rating scale; PASOL: Pain solutions questionnaire; PASS: Pain anxiety symptom scale; PCS: Pre-stratified by degree of pain catastrophizing; PDI: Pain disability index; PHODA: Photo series of daily activities; PIPS: Psychological inflexibility in pain scale; PVAQ: Pain vigilance and attention questionnaire; QALY: Quality adjusted life years; QBPDS: Quebec back pain disability scale; RCT: Randomized controlled trial; SPSS: Statistical program for social science; TIC-P: The trimbos/iMTA questionnaire for costs associated with psychiatric illness; TSK: Tampa scale of kinesiophobia

## Competing interests

The authors declare no competing interests.

## Authors’ contributions

JG developed the project and obtained funding. All authors participated in the final design of the study. JR wrote the first draft of this paper and the other authors provided input. JR and SH are involved in data collection. WR supervises the project. JG, JR and SH will analyse the data. All authors read and approved the final manuscript.
